# A method for patient‐specific DVH verification using a high‐sampling‐rate log file in an Elekta linac

**DOI:** 10.1002/acm2.13849

**Published:** 2022-11-28

**Authors:** Shiro Nishiyama, Akihiro Takemura

**Affiliations:** ^1^ Department of Radiotechnology Saiseikai Kawaguchi General Hospital Kawaguchi Japan; ^2^ Division of Health Sciences, Graduate School of Medical Sciences Kanazawa University Kanazawa Japan; ^3^ Faculty of Health Sciences, Institute of Medical, Pharmaceutical and Health Sciences Kanazawa University Kanazawa Japan

**Keywords:** log file, patient‐specific QA, radiotherapy, volumetric‐modulated arc therapy

## Abstract

We have proposed a method for patient‐specific dose–volume histogram (DVH) verification using a 40‐ms high‐sampling‐rate log file (HLF) available in an Elekta linac. Ten prostate volumetric‐modulated arc therapy plans were randomly selected, and systematic leaf position errors of ±0.2, ±0.4, or ±0.8 mm were added to the 10 plans, thereby producing a total of 70 plans. An RTP file was created by interpolating each leaf position in the HLF to obtain values at each control point, which is subsequently exported to a treatment planning system. The isocenter dose calculated by the HLF‐based plan to a phantom (Delta4 Phantom+) was compared to that measured by the diode in the phantom in order to evaluate the accuracy of the HLF‐based dose calculation. The *D*95 of the planning target volume (PTV) was also compared between the HLF‐based plans and the original plans with the systematic leaf position errors, the latter being referred to as theory‐based plans. Sensitivities of the DVH parameters in the target, the rectum, and the bladder were also calculated with the varied systematic leaf position errors. The relative differences in the isocenter doses between the HLF‐based calculations and the measurements among the 70 plans were 0.21% ± 0.67% (SD). The maximum relative differences in PTV *D*95 between the HLF‐based and the theory‐based plans among the 70 cases were 0.11%. The patient‐specific DVH verification method detected a change in the target DVH parameters of less than 1% when the systematic leaf position error was ±0.2 mm. It is therefore suggested that the proposed DVH verification method may simplify patient‐specific dose quality assurance procedures without compromising accuracy and sensitivity.

## 1 INTRODUCTION

In intensity modulated radiotherapy (IMRT) and volumetric‐modulated arc therapy (VMAT), the accuracy in the leaf positions of the multi‐leaf collimator (MLC) is of critical importance, as inaccurate leaf positions caused by gravity imposed on the leaf carriage system may degrade the dose distributions.^1^ Patient‐specific IMRT or VMAT plan verification prior to treatment is recommended in the International Commission on Radiation Units and Measurements report 83,^2^ and this can be accomplished by absolute dose verification with an ion chamber and dose distribution verification using a film. In addition, a 2D/3D semiconductor detector array and an electric portal imaging device may also be employed.^3^, ^4^ However, these measurement procedures are labor‐intensive. In addition, the pass/fail decision of the measured dose distributions relative to the planned doses is based on mostly 3%/3 mm gamma criteria.^4^ The gamma criteria evaluate the dose discrepancy inside the inspection space without considering dose volume prescriptions and constraints specified in the target and the organs at risk (OARs).

Besides, machine log file analysis was proposed to verify whether treatment delivery accurately reproduced the patient plan.^4^, ^5^, ^6^, ^7^, ^8^, ^9^, ^10^ The Elekta linear accelerator (linac) provides two types of log files with different sampling intervals of 250–500 ms (low‐sampling‐rate log file, LLF) and 40 ms (high‐sampling‐rate log file, HLF). Several studies have been published using log files obtained from the Elekta linac for patient‐specific VMAT quality assurance (QA), but all of them adopted the LLF.^11^, ^12^, ^13^, ^14^, ^15^, ^16^, ^17^, ^18^ Pasler et al. reported linac QA results during VMAT delivery using the LLF.^12^ A large leaf position discrepancy was observed between the log file and the plan at a control point when the leaf speed was the maximum. They concluded that the cause was due to the coarse sampling of the LLF. Katsuta et al. also evaluated the accuracy of patient‐specific VMAT QA using the LLF with a sampling interval of 500 ms, where the minimum systematic leaf position errors of 0.4 mm were employed without showing the impact of smaller systematic leaf position errors.^17^ Szeverinski et al. proposed a secondary Monte Carlo dose calculation for a prostate VMAT plan using the LLF of 250 ms, stating that the log file may bring about deviations at control points because of its larger sampling interval that may require interpolation.^18^ They further mentioned that dose discrepancy was observed in OARs, which was clinically negligible without showing the amounts of the discrepancy. Kabat et al. evaluated MLC performance using a 40‐ms HLF, but nothing was mentioned for patient‐specific QA with the HLF.^19^ Oliver et al. stated that systematic leaf position errors have a greater impact on dose errors than random leaf position errors in VMAT plans.^20^ LoSasso et al. also stated that for dynamic MLC, the MLC opening must be controlled within 0.2 mm to achieve a dose error within 1.0%.^21^ In the above log file references, gamma criteria were always employed for dose verification.

Knowing that the gamma pass rates are not clinically relevant parameters, dose–volume histogram (DVH) parameters for the target and the OARs may be clinically more meaningful for dose verification. On the other hand, Woon et al. reported a sensitivity analysis of DVH parameters using a log file^22^; however, no accuracy analysis was performed. We trust that the accuracy and the sensitivity are equally important to utilize a log file as a means of dose verification.

The purpose of this study was therefore to propose a method for patient‐specific DVH verification using a 40‐ms HLF available in an Elekta linac. Prostate VMAT plans were employed to demonstrate the accuracy and the sensitivity of the proposed method.

## 2 METHODS

In this study, the Elekta Synergy linac with an Agility MLC (Elekta, Stockholm, Sweden) was used to obtain an HLF. The linac control system, Integrity R 4.0 (Elekta, Stockholm, Sweden), writes the leaf position, jaw position, gantry angle, and monitor unit to the HLF every 40 ms.

### 2.1 Patient characteristics and VMAT planning

VMAT plans for randomly selected 10 prostate cancer patients were utilized in this study. The mean age of the patients was 73.5‐year old (range: 66–82). All prostate cancers were of stage I or II, excepting a patient with stage IV. All the patients received the dose to the prostate only and the regional lymph nodes were excluded. A use of the data was approved by the ethical review committee in our hospital with IRB number of 2022‐14. All the VMAT plans were created by Monaco treatment planning system (TPS) version 5.11.02 (Elekta, Stockholm, Sweden). The clinical target volume (CTV) was defined as the volume of the prostate, and the planning target volume (PTV) was defined by adding an isotropic margin of 5 mm to the CTV to account for patient positioning uncertainty. In addition, any portions of the rectum or the bladder were subtracted from the PTV to avoid radiation to those risk organs. The rectum was delineated from the sigmoid colon junction to the anus. The mean volumes and the standard deviations of the CTV, the PTV, the rectum, and the bladder for the 10 patients were 24.66 ± 7.71, 54.05 ± 13.85, 63.10 ± 18.81, and 204.73 ± 58.92 ml, respectively.

The isocenter was positioned at the center of the PTV. All the VMAT plans were configured with 10‐MV photons, a single 360° arc, a collimator angle of 10°, and a dose prescription of 72 Gy in 36 fractions. The calculation grid size was 2 mm, and the dose computation algorithm was X‐ray Voxel Monte Carlo (XVMC) with segment shape optimization. The XVMC calculation results depend on the number of histories and thus the statistical uncertainty. Keall et al. reported that a statistical uncertainty of less than 2% in the maximum dose would not significantly affect isodose lines, DVHs, or biological indices.^23^ We adopted a statistical uncertainty of 0.5% per plan, which was a sufficiently small statistical uncertainty that would not affect the DVHs. The dose constraints for the optimization calculations included a minimum dose of 72 Gy delivered to 95% of the PTV volume, a maximum dose of 70 Gy in the rectum, and a bladder volume of less than 10 ml receiving 60 Gy or above.

### 2.2 Configuring leaf position errors and creating HLF‐based VMAT plans

Systematic leaf position errors of ±0.2, ±0.4, and ±0.8 mm were added to the right side of the active leaf pairs in the beam's eye view that define the radiation fields in the original plan. In this study, the leaf gap width formed by each leaf pair was defined on the isocenter plane, which is referred to as “MLC leaf gap width,” and the minimum leaf gap width on the isocenter plane was constrained to 3.5 mm in the Agility MLC. If the leaf gap width would have gone below this limit, it was adjusted to 3.5 mm.

The plans with six different leaf position errors were created by adding the above systematic errors to the active leaves in the RTP file of the original plan, each of which was further converted to a DICOM‐RT plan file and exported to Monaco TPS for dose calculation. Hereinafter, these plans were referred to as “theory‐based plans” because they were not based on the HLF data.

**FIGURE 1:**
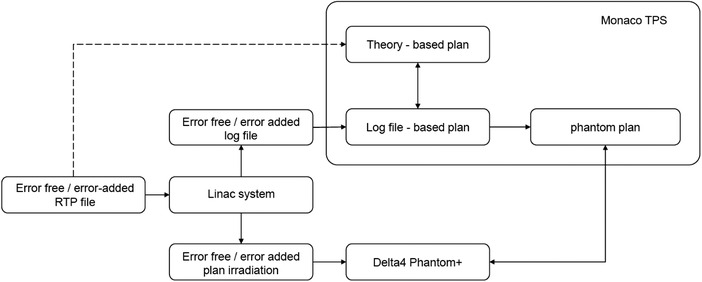
Flowchart of the accuracy tests on high‐sampling‐rate log‐file (HLF)‐based dose calculations, where HLF stands for high‐sampling‐rate log file. Planning target volume (PTV) *D*95 values were calculated in the Monaco treatment planning system (TPS) using the HLF‐based and the theory‐based plans by adding systematic leaf errors to the both plans for comparison. The isocenter doses for the HLF‐based plans were also calculated in the TPS with the Delta4 Phantom+ geometry, which were compared to the measurements by the built‐in detector in the phantom.

The Integrity R 4.0 recorded the linac machine parameters in the HLF (a binary file) every 40 ms, immediately after each beam delivery. An in‐house software was developed using JAVA and Python to obtain the leaf positions at the control points from the HLF and to create DICOM‐RT files in which the leaf positions of the original plans were replaced with those obtained from the HLF. Linear interpolation was further required to obtain values at each control point. The resulting DICOM‐RT files were exported to the Monaco TPS, and the dose distributions were recalculated to obtain the HLF‐based plans under the same conditions as the original plans (Figure 1). As a simple test, an error was added to a single leaf, and the dose was delivered. Then we can check the leaf with errors by analyzing the log file.

### 2.3 Accuracy test by comparing dose distributions between the HLF‐based and the theory‐based plans

PTV *D*95 values were calculated in the Monaco TPS using the HLF‐based and the theory‐based plans for comparison. The systematic leaf errors of 0, ±0.2, ±0.4, and ±0.8 mm were added to the both plans, the errors being added to the right side of the active leaf pairs in beam's eye view. PTV *D*95 is known as a relevant target dose parameter that is associated with tumor local control.

### 2.4 Accuracy test by comparing isocenter doses from the HLF‐based plans with measured isocenter doses

The Delta4 Phantom+ system (ScandiDos, Uppsala, Sweden) was used to measure the absolute dose at the isocenter. This device consists of an array of 1069 p‐Si diodes each having a diameter of 1.0 mm. The Delta4 Phantom+ was calibrated at the time of the linac installation, and the calibration was repeated every 12 months. Before each use of the Delta4 Phantom+, a rectangular field of 10 × 18 cm^2^ was irradiated at gantry angles of 0° and 90° to ensure that the Delta4 Phantom+ was placed at the center of the irradiation field, in such a way that the dose readings of the detectors at the edges of the irradiation field were checked to confirm that the dose values were the same. Using all the 70 theory‐based plans, a prescribed dose of 2 Gy was delivered to the Delta4 Phantom+, and the absolute doses at the isocenter were measured, whereas the HLF was simultaneously acquired for the HLF‐based dose calculation in the TPS.

### 2.5 Sensitivity test of DVH parameters and dose distribution

To evaluate the sensitivity of the HLF‐based plans, the *D*2 (%) values of the PTV and CTV were calculated to assess the hot spots in the target volume, where each of the DVH parameters for the target was normalized to the value calculated by the HLF‐based plan without the leaf position errors. *D*95 (%) and *D*_mean_ (%) were employed to assess the coverage of the PTV and CTV. The values of *D*2 (Gy) of the rectum and bladder were used to assess the hot spots in the OAR volumes, and the *V*70 (ml) and *V*50 (ml) of the rectum and bladder were employed to assess changes in the irradiated volumes of these organs. Based on the HLF‐based plan without leaf position errors, relative changes in the DVH parameters of the HLF‐based plans with systematic leaf position error were calculated. The linear approximation equation and *R* ^2^ were obtained from each of the DVH parameters as a function of the leaf position errors.

## 3 RESULTS

### 3.1 Calculated PTV *D*95 comparison between the HLF‐based and the theory‐based plans

Calculated results and the mean dose differences in the PTV *D*95 values between the HLF‐based and the theory‐based 70 plans are shown with the standard deviations in Table 1. The mean PTV *D*95 values for the HLF‐ and theory‐based plans without the leaf position errors were 72.17 and 72.11 Gy, respectively, with the difference of 0.06 ± 0.04 Gy. The discrepancies of the PTV *D*95 values did not exceed 0.1 Gy in any of the plans with different leaf position errors, and the standard deviations were always <0.05 Gy. The maximum PTV *D*95 differences between an HLF‐based and a theory‐based plan were 0.08 Gy for the leaf position errors of −0.8 and −0.4 mm, whereas a value of 0.1 Gy was equal to 0.14% of the prescribed dose of 72 Gy. The dose differences normalized to the prescribed dose were 0.06% ± 0.09% with a leaf position error of 0.2 mm, and 0.10% ± 0.06% with a leaf position error of −0.2 mm.

**TABLE 1:** Calculated planning target volume (PTV) *D*95 dose comparison between the high‐sampling‐rate log‐file (HLF)‐based and theory‐based 70 plans, when different leaf position errors were added to the right side of the active leaf pairs in beam's eye view.

Leaf position errors (mm)	−0.8	−0.4	−0.2	0.0	+0.2	+0.4	+0.8
Mean HLF‐based plan (Gy)	70.31	71.28	71.74	72.17	72.59	72.99	73.73
Mean theory‐based plan (Gy)	70.22	71.20	71.66	72.11	72.55	72.94	73.66
Mean difference (Gy)	0.08	0.08	0.07	0.06	0.04	0.05	0.06
SD (Gy)	0.04	0.05	0.04	0.04	0.06	0.02	0.03

Abbreviation: SD, standard deviation.

### 3.2 Isocenter dose comparison between the HLF‐based calculation and the measurements

The plans for the 10 patients have 7 different systematic leaf position errors (including the error of 0 mm) added to the right side of the active leaf pairs in the beam's eye view. The isocenter mean dose difference and the standard deviation between the calculation for the 70 HLF‐based plans and the measurements were 0.005 ± 0.015 Gy, and the dose differences normalized to the measurements being 0.21% ± 0.67%.

### 3.3 Sensitivity of DVH parameters when the leaf position errors were varied

Table 2 shows the sensitivities of the calculated DVH parameters (*D*2, *D*95, *D*98, and *D*_mean_ for PTV and CTV; *D*2, *V*50, and *V*70 for rectum and bladder) when the leaf position errors were varied in the HLF‐based plans. The dose errors in the PTV and the CTV were normalized to the doses without the leaf position errors. For all the DVH parameters in the PTV and the CTV, the relative mean dose errors with leaf position errors of ±0.2 mm were less than 1.0%. For the PTV, the largest relative mean error of −2.92% was found for the *D*98 of the HLF‐based plan with a leaf position error of −0.8 mm. For the CTV, the largest relative mean error of 2.05% was found for the *D*2 of the HLF‐based plan with a leaf position error of 0.8 mm. The *D*2 values for the rectum with leaf position errors of +0.2 and −0.2 mm showed mean error of 0.72 and −0.54 Gy, respectively. The *D*2 values for the bladder with leaf position errors of +0.2 and −0.2 mm varied by 0.46 and −0.41 Gy, respectively. In terms of the OAR, the *D*2 with the HLF‐based plan and leaf position error of 0.8 mm showed the largest mean error of 2.72 Gy for the rectum and 1.88 Gy for the bladder. The maximum mean errors in the *V*70 and *V*50 with the leaf position errors up to ±0.8 mm were <0.6 ml for the rectum and <1.2 ml for the bladder.

**TABLE 2:** The sensitivities of the calculated dose–volume histogram (DVH) parameters (*D*2, *D*95, *D*98, and *D*_mean_ for planning target volume [PTV] and clinical target volume [CTV]; *D*2, *V*50, and *V*70 for rectum and bladder) when the leaf position errors were varied in the high‐sampling‐rate log‐file (HLF)‐based plans.

	PTV				CTV			
Leaf errors (mm)	*D*2 (%)	*D*98 (%)	*D*95 (%)	*D*_mean_ (%)	*D*2 (%)	*D*98 (%)	*D*95 (%)	*D*_mean_ (%)
+0.8	2.61 ± 0.58	2.33 ± 0.50	2.25 ± 0.48	2.27 ± 0.51	2.05 ± 0.55	1.59 ± 0.45	1.75 ± 0.77	1.74 ± 0.46
+0.4	1.14 ± 0.18	1.09 ± 0.14	1.05 ± 0.13	1.02 ± 0.15	0.87 ± 0.25	0.73 ± 0.19	0.72 ± 0.19	0.77 ± 0.20
+0.2	0.45 ± 0.24	0.52 ± 0.09	0.49 ± 0.11	0.46 ± 0.11	0.33 ± 0.21	0.33 ± 0.17	0.34 ± 0.19	0.33 ± 0.17
0.0	0.00 ± 0.00	0.00 ± 0.00	0.00 ± 0.00	0.00 ± 0.00	0.00 ± 0.00	0.00 ± 0.00	0.00 ± 0.00	0.00 ± 0.00
−0.2	−0.61 ± 0.13	−0.71 ± 0.10	−0.68 ± 0.11	−0.63 ± 0.11	−0.51 ± 0.21	−0.51 ± 0.20	−0.49 ± 0.19	−0.45 ± 0.21
−0.4	−1.08 ± 0.15	−1.40 ± 0.12	−1.32 ± 0.12	−1.32 ± 0.12	−0.87 ± 0.22	−0.87 ± 0.22	−0.91 ± 0.20	−0.85 ± 0.21
−0.8	−2.02 ± 0.25	−2.92 ± 0.28	−2.73 ± 0.26	−2.35 ± 0.26	−1.53 ± 0.25	−1.53 ± 0.25	−1.87 ± 0.31	−1.67 ± 0.27

	Rectum			Bladder		
Leaf errors (mm)	*D*2 (Gy)	*V*70 (ml)	*V*50 (ml)	*D*2 (Gy)	*V*70 (ml)	*V*50 (ml)
+0.8	2.72 ± 0.36	0.45 ± 0.24	0.53 ± 0.19	1.88 ± 0.37	1.19 ± 0.49	1.10 ± 0.41
+0.4	1.38 ± 0.17	0.22 ± 0.12	0.26 ± 0.09	0.89 ± 0.12	0.58 ± 0.22	0.53 ± 0.18
+0.2	0.72 ± 0.14	0.11 ± 0.06	0.14 ± 0.05	0.46 ± 0.07	0.30 ± 0.12	0.27 ± 0.10
0.0	0.00 ± 0.00	0.00 ± 0.00	0.00 ± 0.00	0.00 ± 0.00	0.00 ± 0.00	0.00 ± 0.00
−0.2	−0.54 ± 0.05	−0.08 ± 0.06	−0.10 ± 0.04	−0.41 ± 0.07	−0.25 ± 0.09	−0.23 ± 0.08
−0.4	−1.24 ± 0.07	−0.15 ± 0.10	−0.21 ± 0.07	−0.80 ± 0.15	−0.51 ± 0.21	−0.48 ± 0.15
−0.8	−2.57 ± 0.17	−0.19 ± 0.14	−0.46 ± 0.14	−1.73 ± 0.28	−1.10 ± 0.46	−1.00 ± 0.32

*Note*: The leaf position errors were added to the right side of the active leaf pairs in beam's eye view. The dose errors in the PTV and the CTV were normalized to the doses without the leaf position errors. The absolute dose errors for the OARs were calculated in reference to those without systematic leaf errors, whereas the changes in irradiated volumes for the OARs were calculated in reference to those volumes without systematic leaf errors.

Abbreviation: OARs, organs at risk.

**FIGURE 2:**
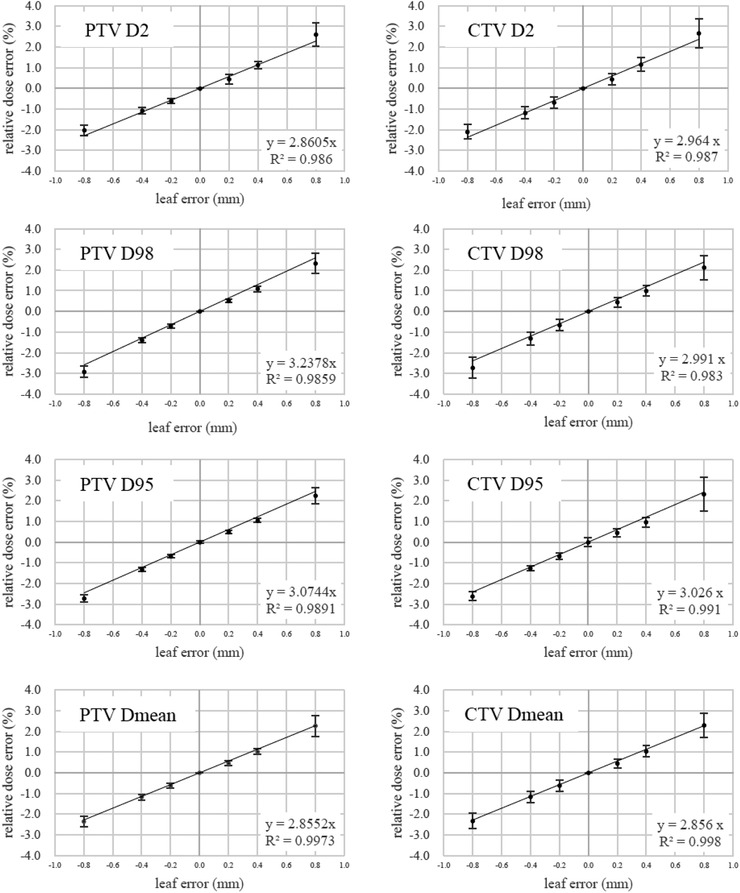
Sensitivity of the target dose–volume histogram (DVH) parameters calculated for the 70 high‐sampling‐rate log‐file (HLF)‐based plans when the different leaf position errors were added to the right side of the active leaf pairs in the beam's eye view. The dose errors (means and SD's) in each plot were normalized to the dose without the leaf position errors. A straight line giving the least squared errors was calculated using the data shown in each plot, and by definition, the line always crosses the origin. The calculation was performed by Microsoft Excel. CTV, clinical target volume; PTV, planning target volume.

**FIGURE 3:**
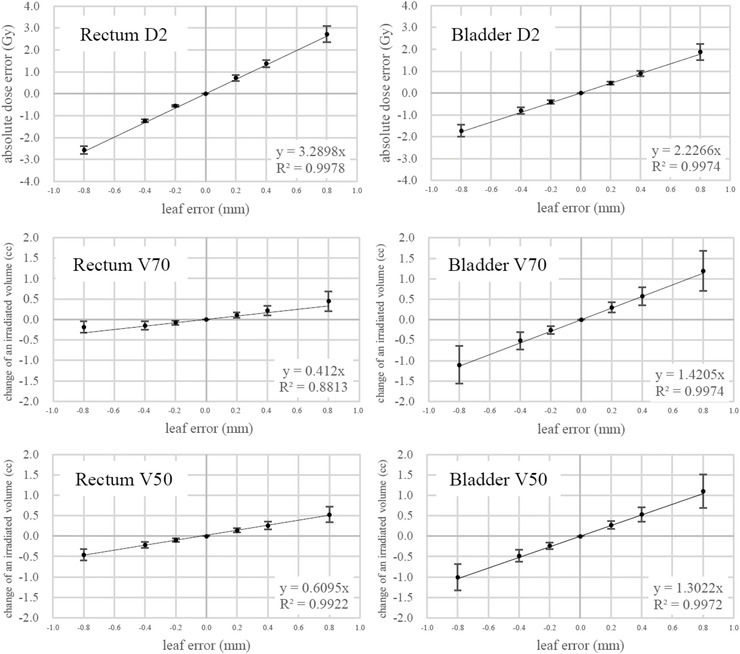
Sensitivity of the rectum and the bladder dose–volume histogram (DVH) parameters calculated for the 70 high‐sampling‐rate log‐file (HLF)‐based plans when the different leaf position errors were added to the right side of the active leaf pairs in the beam's eye view. The absolute dose errors were calculated in reference to the doses without systematic leaf errors, whereas the changes in irradiated volumes were calculated in reference to those volumes without systematic leaf errors. Again, a straight line giving the least squared errors was calculated using the data shown in each plot, and by definition, the line always crosses the origin. The calculation was performed by Microsoft Excel.

In Figures 2 and 3, the DVH parameters changed almost linearly with the leaf position errors, with *R* ^2^ ranging from 0.88 to 1.00. The minimum *R* ^2^ was found in the *V*70 of the rectum. By employing linear approximation as shown in each plot of these figures, we were able to calculate the sensitivities of the DVH parameters per unit leaf displacement. The sensitivities of the *D*_mean_ for the PTV and the CTV were both 2.86%/mm, whereas those of the *D*95 values for the PTV and the CTV were 3.07 and 3.03%/mm, respectively. Those of the *D*2 values for the rectum and the bladder were 3.29 and 2.23 Gy/mm, respectively.

**FIGURE 4:**
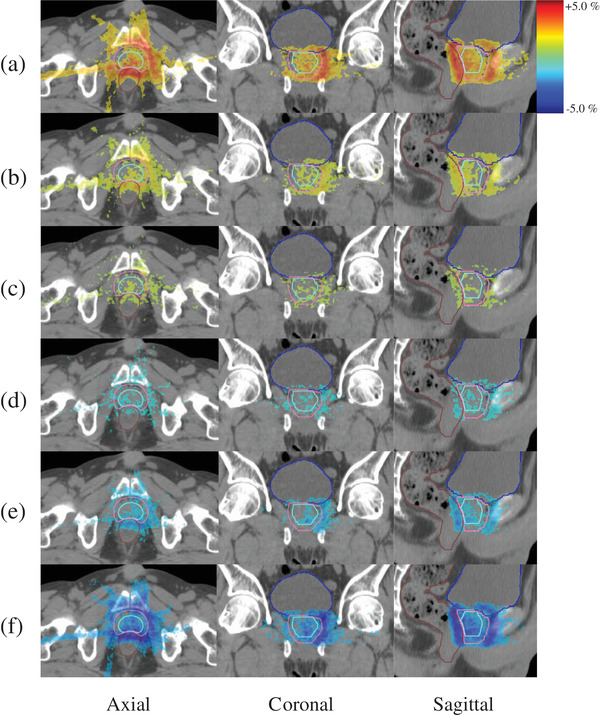
A case presentation of areas on the three orthogonal planes showing significant dose differences between the high‐sampling‐rate log‐file (HLF)‐based plans with and without systematic leaf position errors, when the leaf position errors of (a) 0.8, (b) 0.4, (c) 0.2, (d) −0.2, (e) −0.4, and (f) −0.8 mm were added to the right side of the active leaf pairs in the beam's eye view. The results in (c) and (d) show the impacts on the rectal wall doses even when small errors of ± 0.2 mm were added.

Figure 4 is a case presentation of areas on the three orthogonal planes showing significant dose differences between the HLF‐based plans with and without the systematic leaf position errors, when the leaf position errors of (a) 0.8, (b) 0.4, (c) 0.2, (d) −0.2, (e) −0.4, and (f) −0.8 mm were added to the right side of the active leaf pairs in the beam's eye view. The positive leaf error displaced the right‐side active leaves toward the right direction in beam's eye view, whereas the negative leaf error displaced them toward the left. The red region shows that the dose was higher with the added leaf position errors, whereas the blue region shows that the dose was lower with the added leaf position errors. The larger the leaf position errors, the larger the dose differences. It was also demonstrated that the impact on the dose distribution was visualized even with a systematic leaf position error of 0.2 mm.

## 4 DISCUSSION

In this study, we have evaluated if the proposed method may simplify patient‐specific dose QA procedures without compromising accuracy and sensitivity. As to accuracy, the maximum mean difference in the PTV *D*95 between the HLF‐ and theory‐based 70 plans with different leaf position errors up to ±0.8 mm was 0.08 Gy as shown in Table 1, which is 0.11% after normalization to the prescribed dose of 72 Gy. It is therefore suggested that the DVH parameter verification may be feasible using the HLF with an accuracy less than 0.2%. For sensitivity, the relative differences in the isocenter doses between the HLF‐based calculations and the measurements by the Delta4 Phantom+ among the 70 plans were 0.21% ± 0.67% (SD). The uncertainty of the Delta4 detector response was reported to be 0.1%,^24^ whereas Monaco TPS has a calculation uncertainty of 0.5%. Consequently, the HLF‐based calculations could reproduce the measured isocenter doses within calculation precision.

Oliver et al. added leaf position errors to the DICOM‐RT files of head‐and‐neck VMAT plans created on an Eclipse TPS (Varian Medical Systems, Palo Alto, CA) to evaluate a log‐file‐based plan and concluded that the leaf position error should be within 0.6 mm in order for the PTV mean dose change to be within 2%.^20^ We found that the leaf position error should be within 0.65 mm to satisfy the 2% difference for the PTV mean dose (Figure 2). This leaf position error is comparable to the value Oliver et al. reported, but slightly larger than theirs. This may be explained by the difference in the mean MLC gap between head‐and‐neck VMAT plans and prostate VMAT plans, as it is known that the dose sensitivity increased when the MLC gap was decreased.^25^ LoSasso et al. recommended that the leaf position error of a dynamic MLC should be less than 0.2 mm in the isocenter plan to keep the dose error below 1.0%.^21^ In our study, it was shown in Table 2 that the PTV *D*95 changed by 0.49% and −0.68% with the systematic leaf position errors of 0.2 and −0.2 mm, respectively, both of which were below 1.0%.

Figure 3 shows that *V*70 and *V*50 of the bladder had larger sensitivities compared to those of the rectum when the leaf position errors were varied. The present authors trust that this is because the bladder volume is larger than the rectum volume, and therefore, the *V*70 and the *V*50 of the bladder are also larger than those of the rectum. When a small systematic leaf position error was added to each active leaf, isodose surfaces of 70 and 50 Gy were slightly deformed. Then, *V*70 and *V*50 were slightly changed in proportion to the original values of *V*70 and *V*50, respectively.

Even with the systematic leaf position errors of ±0.2 mm, the HLF‐based plan detected the changes in the *V*70 and *V*50 in the rectum and the bladder, regardless of the OAR volumes.

Figure 4 shows significant differences in dose distributions between the HLF‐based plans with and without leaf position errors around the PTV, and the dose errors were visualized even when the leaf position error was ±0.2 mm. In other words, the HLF‐based plan could detect the target dose difference when the leaf position errors were ±0.2 mm.

We have discussed the accuracy and the sensitivity of the proposed DVH verification method. In practice, action levels need to be defined for each DVH parameters. Yi et al. employed a change of 5% as the action levels^26^; however, more studies are awaited to establish the action levels of each DVH parameters from a clinical point of view.

This study is subject to the limitation that we used only VMAT plans for prostate cancer, and the accuracy of our QA method should be investigated for other delivery methods and other tumor sites. In general, log file analysis does not consider beam output variations, MLC leaf transmissions and MLC tongue and groove effects. These characteristics are not patient‐specific, and therefore, linac machine QA can take care of them along with a TPS. For example, MLC leaf transmission can be modeled in the TPS. The tongue and groove effect can be reduced by slightly rotating the collimator such as by 10°.

## 5 CONCLUSION

We have proposed a new method for patient‐specific DVH verification using a 40‐ms HLF available in an Elekta linac. The accuracy and sensitivity were validated through various calculations and measurements. It is therefore suggested that the proposed DVH verification method may simplify patient‐specific dose QA procedures without compromising accuracy and sensitivity.

## AUTHOR CONTRIBUTIONS

Shiro Nishiyama conceived of the presented idea and carried out the experiment. In addition, Shiro Nishiyama developed the in‐house software for analyzing the data and wrote the manuscript with support from Akihiro Takemura.

## CONFLICTS OF INTEREST

No conflicts of interest.
